# A Model of Waardenburg Syndrome Using Patient-Derived iPSCs With a *SOX10* Mutation Displays Compromised Maturation and Function of the Neural Crest That Involves Inner Ear Development

**DOI:** 10.3389/fcell.2021.720858

**Published:** 2021-08-06

**Authors:** Jie Wen, Jian Song, Yijiang Bai, Yalan Liu, Xinzhang Cai, Lingyun Mei, Lu Ma, Chufeng He, Yong Feng

**Affiliations:** ^1^Department of Otorhinolaryngology, Xiangya Hospital Central South University, Changsha, China; ^2^Province Key Laboratory of Otolaryngology Critical Diseases, Changsha, China; ^3^Department of Geriatrics, National Clinical Research Centre for Geriatric Disorders, Xiangya Hospital, Central South University, Changsha, China; ^4^Department of Otorhinolaryngology, The Affiliated Changsha Central Hospital, Hengyang Medical School, University of South China, Changsha, China

**Keywords:** Waardenburg syndrome, SOX10, induced pluripotent stem cells (hiPSC), neural crest cells (NCCs), inner ear development, transcriptome analysis

## Abstract

Waardenburg syndrome (WS) is an autosomal dominant inherited disorder that is characterized by sensorineural hearing loss and abnormal pigmentation. *SOX10* is one of its main pathogenicity genes. The generation of patient-specific induced pluripotent stem cells (iPSCs) is an efficient means to investigate the mechanisms of inherited human disease. In our work, we set up an iPSC line derived from a WS patient with *SOX10* mutation and differentiated into neural crest cells (NCCs), a key cell type involved in inner ear development. Compared with control-derived iPSCs, the *SOX10* mutant iPSCs showed significantly decreased efficiency of development and differentiation potential at the stage of NCCs. After that, we carried out high-throughput RNA-seq and evaluated the transcriptional misregulation at every stage. Transcriptome analysis of differentiated NCCs showed widespread gene expression alterations, and the differentially expressed genes (DEGs) were enriched in gene ontology terms of neuron migration, skeletal system development, and multicellular organism development, indicating that *SOX10* has a pivotal part in the differentiation of NCCs. It’s worth noting that, a significant enrichment among the nominal DEGs for genes implicated in inner ear development was found, as well as several genes connected to the inner ear morphogenesis. Based on the protein-protein interaction network, we chose four candidate genes that could be regulated by *SOX10* in inner ear development, namely, *BMP2*, *LGR5*, *GBX2*, and *GATA3*. In conclusion, SOX10 deficiency in this WS subject had a significant impact on the gene expression patterns throughout NCC development in the iPSC model. The DEGs most significantly enriched in inner ear development and morphogenesis may assist in identifying the underlying basis for the inner ear malformation in subjects with WS.

## Introduction

Waardenburg syndrome (WS) is a rare autosomal dominant inherited disorder. WS is distinguished by sensorineural hearing loss (SNHL) and pigment abnormalities, such as hypo-pigmentation of the skin, a white forelock, premature graying, or heterochromia iridum (Waardenburg, 1951). There are four WS subtypes categorized by the presence or lack of other clinical symptoms. Clinically, WS1 and WS2 are the most frequently noted (Dourmishev et al., 1999). The actual incidence of WS is thought to be 1/42,000, and may account for 2–5% of congenital deafness (Nayak and Isaacson, 2003). Several mutations in six genes have thus far been reported to be linked to WS, including *PAX3*, *MITF*, *SOX10*, *EDNRB*, *EDN3*, and *SNAI2* (Pingault et al., 2010). Researchers have proposed several explanations for the clinical characteristics of WS. At present, the theory of neural crest hypoplasia is the most widely noted. This theory holds that the embryonic neural crest is the source of melanocytes, frontal bone, limb muscles, and intramural ganglia, and that their dysfunction due to WS impacts different tissues and organs, leading to a series of abnormalities (Bolande, 1997; Knecht and Bronner-Fraser, 2002).

As the inner ear forms and develops, neural crest cells move from rhombomere 4 to the otocyst and begin to differentiate into glial cells of the cochleovestibular ganglion and intermediate melanocytic cells of the cochlear stria vascularis, both of which are essential cell types in the inner ear (Tachibana et al., 2003; Freter et al., 2013; Kim et al., 2013). Recently, a few studies have demonstrated that neural crest cells (NCCs) also participate in the development of the inner ear neurosensory components, which are thought to be lineages derived from the otocyst. However, the contributions of NCCs to the neurosensory components of the inner ear are not completely understood (Freyer et al., 2011; Mao et al., 2014; Karpinski et al., 2016).

SOX10 is a key transcription factor during the development of the neural crest. In addition, SOX10 has a pivotal part in maintaining the pluripotency, survival, and proliferation of NCCs (Southard-Smith et al., 1998). *SOX10* mutations are primarily connected to the pathogenesis of WS2 and WS4 (Bondurand et al., 2007; Chen et al., 2010). In addition, *SOX10* mutations can induce Kallmann syndrome (KS, OMIM 308700) as well a plethora of neurological symptoms in the neural crest (PCWH), such as outer peripheral demyelinating neuropathy, central myelination disorder, WS, and Hirschsprung’s disease (HD) (Pingault et al., 2000, 2013; Inoue et al., 2004). Previous studies have demonstrated that WS subjects with *SOX10* mutations more frequently exhibit different degrees of inner ear deformities. Nevertheless, additional research is needed to elucidate the target genes and pathways regulated by SOX10 in inner ear development (Breuskin et al., 2009; Elmaleh-Bergès et al., 2013).

Human-induced pluripotent stem cell (iPSC) technology is a new tool for researching human developmental disorders. Genotype-specific molecular and cellular phenotypes that occur throughout differentiation can be modeled by these cells. By reprogramming somatic cells obtained from subjects into a state resembling embryonic stem cells and then differentiating them into disease-relevant cell types, researchers can use iPSC technology to produce an almost unlimited source of human tissues with the genetic mutations found at the genesis of the disease. This technology is a powerful tool that can be used to derive patient-specific cells for human disease modeling. In addition, iPSC technology is promising for personalized cell therapies (Takahashi et al., 2007; Tang et al., 2020; Zhang et al., 2020a). It is currently thought that a global disturbance of transcriptional regulation due to SOX10 deficiency, which is still not fully understood, may be one cause of the aberrant phenotypes found in WS patients (Huang et al., 2021). Because SOX10 functions as a DNA-binding protein, the likelihood that SOX10 may directly modulate transcription in the nucleus is high. In WS patients with SOX10 mutations, no microarray-based gene expression profiling data were generated. RNA-seq analysis is urgently needed to fully reveal the transcriptional perturbation induced by SOX10 deficiency.

In the present study, we provide details about a Chinese patient with WS2, and noted a *de novo* heterozygous mutation in *SOX10*. Patient-derived fibroblasts were gathered to produce iPSCs, and we then differentiated these iPSCs into NCCs *in vitro*, and contrasted their differentiation potential with iPSCs derived from a normal healthy patient to examine disorders linked to this syndrome. Further, we completed transcriptomics analysis of the differentiating cells throughout the *in vitro* differentiation process to examine the underlying genetic basis of WS. The genes that we characterized as relevant for NCC differentiation and development will assist in the discovery of new therapies for WS. In this work, we generated a research model and offer insights for additional studies on the mechanism(s) governing WS.

## Materials and Methods

### Ethics Statement

The Xiangya Ethics Committee approved the protocol for this study, and signed informed consent was provided by every donor before sample collection. The laboratory research on the derivation and use of human iPSC lines was approved by the Ethics Committee of Xiangya Hospital Central South University (XHCSU) in accordance with local regulations, and all of the animal experiments were conducted based on XHCSU ethical guidelines.

### Clinical Evaluation

The proband was recruited from the Otology Clinic at XHCSU. Other family members were included, along with 100 controls comprised of unselected, unrelated, and sex-matched healthy individuals. Comprehensive clinical history, audiologic, neurologic, ophthalmologic, and dermatologic examinations were conducted on proband and all family members. The audiologic and neurologic examinations consisted of otoscopy, pure-tone audiometry (PTA), immittance, distortion product otoacoustic emission (DPOAE), and auditory brain-stem response (ABR) tests. Another auditory steady-state response (ASSR) test was conducted for those patients who did not do well with the PTA test because of their young age (II-1 and II-2). Special attention was paid to pigmentary alterations in the skin, hair, and iris—as well as additional developmental defects, such as dystopia canthorum and limb abnormalities. The degree of hearing loss was defined based on the ASSR and three frequencies: 500, 1,000, and 2,000 Hz. Hearing loss was categorized as follows: a normal hearing level (HL) at < 26 dB (decibels); mild HL, 26–40 dB; moderate HL, 41–70 dB; severe HL, 71–90 dB; and profound HL, > 90 dB.

### DNA Extraction and Mutational Analysis

Genomic DNA was removed from peripheral blood samples of the subjects and healthy controls according to the standard procedure. Whole genomic DNA was isolated with a TIANamp Blood DNA Kit (Tiangen Biotech, China.) and quantified with an ultraviolet spectrophotometer Du800 (Beckman Coulter, United States). The DNA was then kept at –20°C until use. PCR and Sanger sequencing was conducted on each of the coding exons and flanking splicing sites of the WS-related genes, including *MITF*, *SOX10*, *PAX3*, *EDNRB*, *EDN3*, and *SNAI2*. The PCR products were treated with shrimp alkaline phosphatase and exonuclease-I to degrade deoxynucleotide triphosphates and unincorporated PCR primers. The purified amplicons were combined with 10 picomoles of the forward and reverse PCR primers for bidirectional sequencing on an ABI-Prism 3100 DNA sequencer via dye-termination chemistry (Applied Biosystems, United States), and the SeqMan II program (DNA-STAR, United States) was utilized to compare results. Once the mutation was determined, DNA samples from related family members and controls were then screened for the identical mutation.

### Collection and Establishment of Fibroblast Cultures From Skin Tissue of a WS Patient

After obtaining written informed consent from the donor, human skin samples were collected from the proband (WS patient). The biopsy tissue was put in a sterile tube filled with phosphate-buffered saline (PBS) containing 1% penicillin/streptomycin (Invitrogen, United States), and kept at 4°C. The steps that follow were performed in a tissue culture hood under aseptic conditions and using sterile instruments.

The subcutaneous fat and capillaries were completely removed from the sample tissue, and the tissue was moved to a 50-ml Falcon tube containing 4 ml of 0.05% trypsin/EDTA (Invitrogen, United States) and incubated overnight at 4°C. The epidermis was manually extracted from the tissue, and the supernatant was discarded after adding 4 ml of freshly-prepared fibroblast culture medium [DMEM containing 10% FBS, 1% penicillin/streptomycin, 1% glutamine, and 1% non-essential amino acids (Invitrogen, United States)]. The dermal tissues were dissected into small pieces, placed in a 100-mm Petri dish, and incubated at 4°C in 5% CO_2_ for 3 h to allow the tissues to adhere to the bottom of the dish. Two milliliters of fibroblast culture medium were added to cover the bottom and ensure that the pieces stay moist. The tissues were incubated at 37°C in 5% CO_2_, and 3 ml of fibroblast culture medium was put in on the following day; the medium was subsequently changed every 3 day. An optical microscope was used to monitor the cultures daily. The tissues were carefully removed when dense outgrowths of fibroblasts appeared, the medium was aspirated, and fresh culture medium was added to maintain the growing fibroblasts (in passage 1). The cells were passaged with trypsin/EDTA at a ratio of 1:3 until the cells reached 80% confluency. Cells from passages 3–5 were then utilized for the induction of iPSCs.

### Generation and Culture of iPSCs

The primary fibroblasts were cultured in hFib medium at 37°C in 5% CO_2_. The fibroblasts (5 × 10^5^ cells) were electroporated with 0.5 μg per vector of five episomal vectors (pCXLE-hUL, pCXLEhOCT3/4-shp53-F, pCXLE-hSK, pCXWB-EBNA1, and pCXLE-EGFP) in order to produce the iPSCs. Electroporation was conducted with the Basic Nucleofector^TM^ Kit for Primary Mammalian Epithelial Cells (Lonza, Switzerland) and the Lonza Nucleofector^TM^ 2b device, program X-005. Following electroporation, the cells were seeded on gelatin-coated 100-mm dishes cultured in hFib medium with the addition of 0.5 mM sodium butyrate (Sigma, United States) and 50 μg/ml VitC (Sigma, United States). The medium was emptied and refilled daily. After 8 day, the cells were moved to Matrigel (Corning)-coated six-well plates at a density of 5 × 10^4^ cells/cm^2^ and cultured in mTeSR medium (Stem Cell Technologies). Two days after the transfer, 10 μM Y-27632 (ROCK inhibitor) was added, and the medium was emptied and refilled on alternating days. The iPSC colonies were manually removed and cultured in mTeSR on Matrigel-coated 24-well plates after 14–21 days. Accutase (Gibco, United States) was used to passage the iPSCs every 6 d at a 1:6 split ratio using, and the iPSCs were kept at 37°C in a 5% CO_2_ incubator (Thermo Fisher Scientific, United States).

### Induction of Neural Crest Cells (NCCs) From iPSCs

The differentiation of iPSCs into NCCs was completed according to the standards detailed prior (Chambers et al., 2011). In short, embryoid bodies were generated in EB Medium (KO-DMEM supplemented with 20% KO-Serum Replacement, 1% GlutaMax-I, and 1% non-essential amino acids) with 500 nM LDN193189 (Stemgent, United Kingdom) and 10 μM SB431542 (Tocris Bioscience, United Kingdom) for 3 day. Culture was carried out in EB Medium supplemented with 2% N-2 (Life Technologies), 1% GlutaMax-I, 100 nM EDN3, 25 ng/ml BMP4, and 50 ng/ml stem cell factor (SCF) (R&D Systems, United States) for the next 3 day. On day 6, embryoid bodies were attached to feeder-free fibronectin-coated culture flasks in Neurobasal Medium supplemented with 2% B-27, 1% N-2, 1% GlutaMax-I, 100 nM EDN3, 25 ng/ml BMP4, and 50 ng/ml SCF. The cells that grew were fed on alternating days for maintenance and expansion until differentiation occurred (day 12). From day 2 and 12 onward, 3 μM CHIR99021 (Stemgent, United Kingdom) was added to the medium.

### Quantitative Reverse Transcription-Polymerase Chain Reaction (qRT-PCR)

RNA was removed from samples using Trizol reagent (Sangon, China) following the company’s directions, and 1 μg of RNA was reverse-transcribed utilizing the PrimeScript^TM^ II 1st Strand cDNA Synthesis Kit (Takara, Japan). All of the qRT-PCR analyses were performed on a Step One plus Real-Time PCR System (ABI) with 2 × SYBR Master Mix (Yeasen, China). The relative expression levels of the target genes were calculated using the 2^–ΔΔCt^ method, and GAPDH was utilized as the internal control (the primers are shown in Supplementary Table 1). Each experiment was repeated thrice, and the average value was taken as the experimental result. The statistical significances for all of the RT-qPCR data were analyzed with unpaired Student’s *t*-tests.

### Western Blot (WB)

Cell extracts that were representative of three independent experiments were prepared from NCCs in a SOX10 mutant and a normal control, and the extracted proteins were analyzed. The antibodies used for Western blot included rabbit anti-SOX10 (Abcam, United Kingdom), mouse anti-GAPDH (Good Here, AB-M-M001) as a primary antibody, HRP-labeled Goat Anti-Rabbit IgG (Beyotime, China), and HRP-labeled Goat Anti-Mouse IgG (Beyotime, China) as a second antibody.

### Alkaline Phosphatase (AP) Staining

An AP Staining Kit (Beyotime, China) was used to assess alkaline phosphatase (AP) activity following the manufacturer’s protocol. The images were assessed using a Nikon 300 inverted confocal microscope.

### Immunofluorescence Staining

The iPSCs were fixed in 4% paraformaldehyde for 20 min at room temperature and then permeabilized using 1% Triton X-100 (Sigma, United States) for 10 min. Following blocking with 5% bovine serum albumin (BSA) (Sangon, China) for 1 h at room temperature, the samples were incubated overnight with the primary antibodies in PBS solution with 5% BSA at 4°C. The next day, secondary antibodies were incubated at room temperature for 1 h. DAPI (Beyotime, China) was used for nuclear counterstaining, and images were observed and photographed using an Olympus confocal microscope and camera. Details about the antibodies are shown in Supplementary Table 2.

### Teratoma Assay

The iPSCs (1 × 10^7^ cells) were gathered and injected subcutaneously into the dorsal flanks of 8-week-old male nude mice (Charles River, China). Approximately 8–10 weeks after injection, teratomas had formed. They were then dissected and fixed in 4% paraformaldehyde, and then embedded in paraffin. Tissue sections were stained using hematoxylin and eosin.

### RNA Sequencing

Two stages of triple replicates (three independent inducing from one source of iPSC) from two samples were obtained (iPSCs and induced neural crest cells (iNCCs) from the normal control and the *SOX10* mutant) for extracting total RNA for further analysis. Total RNA was extracted and RNA integrity was evaluated using the RNA Nano 6000 Assay Kit of the Bioanalyzer 2100 system (Agilent Technologies, United States). One microgram of RNA per sample was utilized for cDNA library preparation with the NEBNext^®^ Ultra^TM^ RNA Library Prep Kit from Illumina^®^ and processed according to the manufacturer’s directions. The library quality was evaluated with the Agilent Bioanalyzer 2100 system. The library preparations were sequenced on an Illumina Novaseq platform, and 150 bp paired-end reads were generated. After being checked for quality control, sequencing reads were mapped to the reference genome with Hisat2 v2.0.5 (Kim et al., 2015), and the raw data were deposited into the GEO database (No. GSE176101).

### Bioinformatic Analysis of RNA-Seq

The raw reads were cleaned by removing reads that had adapters, reads that contained poly-N, and reads of low quality. The resulting clean reads were aligned to the reference genome using Hisat2 v2.0.5, and FeatureCounts v1.5.0-p3 (Liao et al., 2014) was used to quantify the read numbers mapped to every gene and calculate the per kilobase of exon per million fragments mapped (FPKM) to every gene. The differentially expressed genes (DEGs) were analyzed with the DESeq2 method using the online tool NetworkAnalyst 3.0^1^ (Zhou et al., 2019). DEGs had an adjusted *P*-value < 0.05 and | log2 (fold-change) | > 1. Gene ontology (GO) and Kyoto Encyclopedia of Genes and Genomes (KEGG) pathway-enrichment analyses of all of the DEGs were conducted with the online tool DAVID v6.8 (Huang da et al., 2009). GO terms and KEGG pathway terms with an adjusted *P*-value of < 0.05 were considered to be significantly enriched. The protein-protein interaction (PPI) was analyzed using STRING v11.0^2^ (Szklarczyk et al., 2019), with the *SOX10* gene and DEGs uploaded onto STRING with the minimal interaction score set to > 0.4. Cytoscape 3.6.1 software was used to construct the PPI network.

### Statistical Analyses

Data are reported as the mean ± standard deviation (SD) of independent experiments. Statistical analyses were conducted using the Wilcoxon signed-rank test or a one-way analysis of variance (ANOVA) with Prism Graphic software. *P* < 0.05 was considered to be statistically significant.

## Results

### Clinical Findings

**FIGURE 1:**
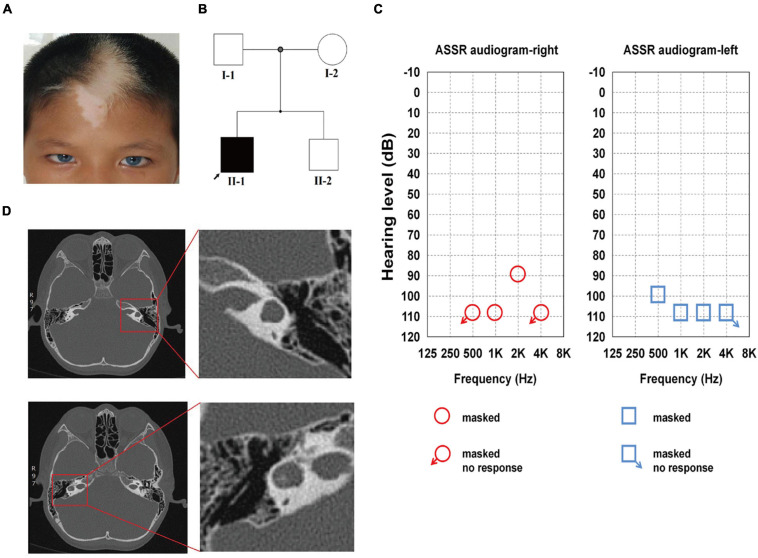
Family history and clinical features of the proband. **(A)** The proband showed bilateral brilliant blue irides, patchy depigmented areas on forehead and white forelock. **(B)** The pedigree indicates that only Family II-1 had the WS-associated phenotypes, which are marked in black. **(C)** ASSR of the left ear: 100, 110, and 110 dB at 0.5, 1, and 2 kHz, respectively, the other frequencies showed no response. ASSR of the right ear: 110 and 90 dB at 1 and 2 kHz, respectively, the other frequencies showed no response. **(D)** Enlarged vestibule and semicircular canal abnormalities on both sides are shown on high-resolution axial CT in the red square.

The proband was 9 years of age and showed brilliant blue bilateral irides, patchy depigmented areas on his forehead, and a white forelock since birth (Figure 1A). The proband was unresponsive to external audio stimuli and unable to speak. Ear injury, otitis media, and contact with ototoxic drugs were not detected. Skin depigmentation was noted, eyesight and intelligence were normal, there was no dystopia cantorum (the W index < 1.95), and no digestive system or skeletal muscle abnormalities were observed. His parents and brother had no pigmentary abnormalities in their skin, hair, or eyes, and they showed no other WS-associated phenotype (Figure 1B).

The audiologic examination of the proband revealed profound bilateral sensorineural HL: there were no bilateral otoacoustic emissions and all of the bilateral ABR thresholds were over 105 dB nHL (the thresholds for ASSR for both ears are shown in detail in Figure 1C). Temporal bone CT scans revealed an enlarged vestibule on either side, left horizontal semicircular canals fused with the vestibule, and right horizontal semicircular canals enlarged and shortened; there were no obvious abnormalities in the shape and size of the bilateral cochleae (Figure 1D). The proband was diagnosed with WS2 based on the WS diagnostic criteria (Liu et al., 1995).

### Identification of Mutations and Pathogenicity Analysis

**FIGURE 2:**
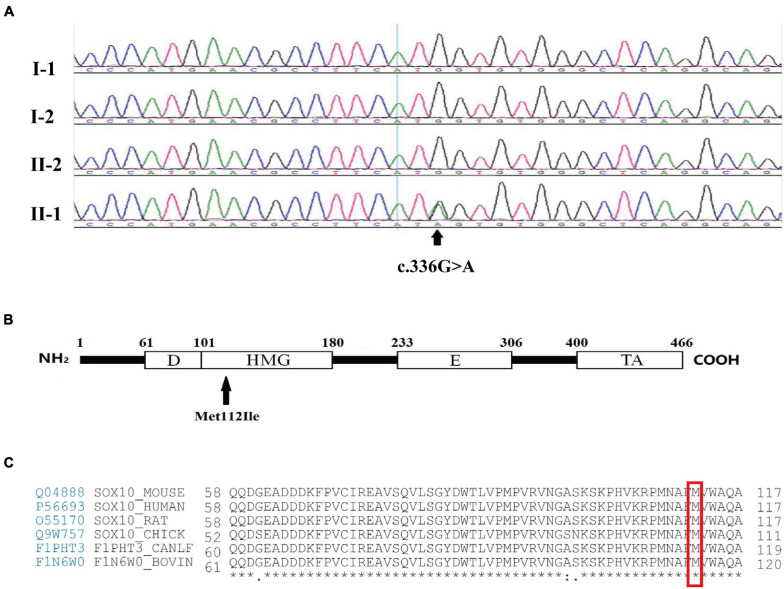
Mutation analysis and amino acid coding diagram. **(A)** DNA sequencing profile revealed that the *SOX10* mutation c.336G > A (p.Met112Ile) was found in the proband (II-1) and not in his father (I-1), mother (I-2), or brother (II-2). The arrow indicates the area of the base substitution. **(B)** Schematic diagram of the *SOX10* gene. The black arrow indicates the mutation site. D, Dimerization domain; HMG, high mobility group domain; E, Conserved domain of SOX8/9/10; TA transactivation domain. **(C)** Protein sequence alignment of vertebrate *SOX10*; note the highly conserved Met112 residues across various species. * represents the same amino acid at the same site among different species.

Following screening for all of the WS-related and congenital hearing loss disease-causing genes, the proband was found to carry a heterozygous mutation of guanine (G) to adenine (A) in position 336 (c.336G > A) of the third exon of *SOX10*. This led to a substitution of the 112th codon (p.Met112Ile). Based on the standards and the guidelines of the American College of Medical Genetics and Genomics (ACMG), this variant is considered pathogenic and was initially identified by Chaoui et al. (2011). Mutations were not found in 100 unrelated healthy control subjects. The proband’s parents and brother had normal phenotypes and carried no corresponding mutations as determined by Sanger sequencing, demonstrating that the mutations occurred *de novo* (Figures 2A,B). No further mutations connected to WS were determined in the proband. Intriguingly, the Met112 residues in SOX10 are highly conserved across various vertebrate species (Figure 2C), indicating the functional importance of this amino acid (Scheithauer et al., 1988).

### iPSCs Derived From an Idiopathic WS Patient With a *SOX10* Mutation Were Generated and Characterized

To better understand the pathogenic mechanism subserving WS, we established an iPSC line from dermal fibroblasts from the proband with the *SOX10* mutation using previously described methods. We also established one normal control iPSC line from an unrelated healthy individual.

**FIGURE 3:**
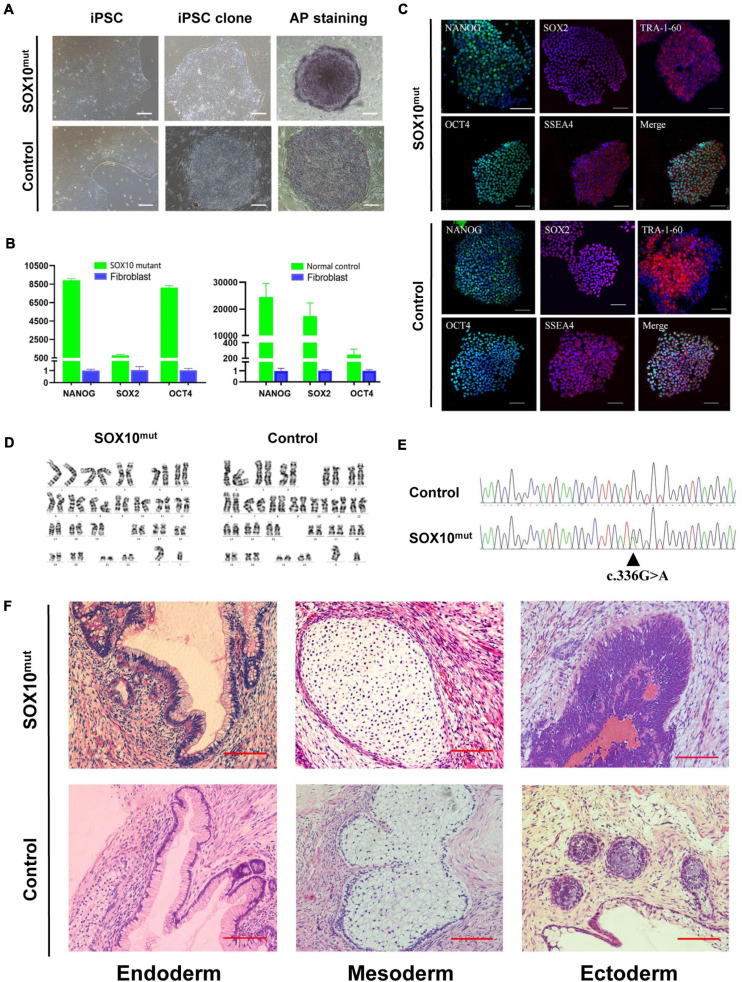
Induction and characterization of *SOX10* mutant and normal control iPSCs. **(A)** The *SOX10* mutant and normal control iPSC clones with typical embryonic stem cell-like and positive alkaline phosphatase staining. Bar, 100 μM. **(B)** qPCR analysis of pluripotency markers in both iPSC lines showed significantly upregulated expression of OCT4, SOX2 and NANOG, in contrast to fibroblasts. **(C)** Immunofluorescence staining in both iPSC lines showed expression of pluripotency markers OCT4, NANOG, TRA-1-60, SOX2, and SSEA-4. Bar, 100 μM. **(D)** Karyotyping analysis showed normal chromosomal structure and numbers in both iPSC lines. **(E)** Sanger sequencing confirmed the mutation in *SOX10* in iPSC lines. **(F)** H&E stainings of teratomas generated from subcutaneous injection of both iPSC lines in NOD/SCID mice. Tumor sections represent differentiated structures as noted. Bar, 100 μM.

Both the *SOX10* mutant and normal control iPSC lines exhibited a typical pluripotent stem cell-like morphology and grew as compact colonies with clearly defined borders and edges. The cells had large nuclei, prominent nucleoli, and a high nuclear-to-cytoplasmic ratio (Figure 3A). We confirmed that the *SOX10* mutant and normal control iPSC lines expressed endogenous pluripotent genes to a high degree as measured by qPCR (Figure 3B). In addition, immunocytochemistry was conducted to investigate the expression of stem cell markers at the protein level. These cells were found to be positive for nuclear (OCT3/4, NANOG, and SOX2) and surface (SSEA4 and TRA1-60) markers of pluripotency, in addition to staining for AP (Figures 3A,C). We then examined the differentiation potential of these iPSC lines. Both lines had the ability to differentiate into the three germ layers (ectoderm, mesoderm, and endoderm) in the teratoma assay (Figure 3F); these iPSC lines presented a normal karyotype (Figure 3D). These findings indicated that the reprogramming of the fibroblasts caused no alterations in the chromosomal or genetic markers. Furthermore, genotyping confirmed the expected compound heterozygous *SOX10* mutation (c.336G > A) in the iPSC line from the WS2 patient (Figure 3E).

### *SOX10* Deficiency Results in Altered Gene Expression Patterns in iPSCs

**FIGURE 4:**
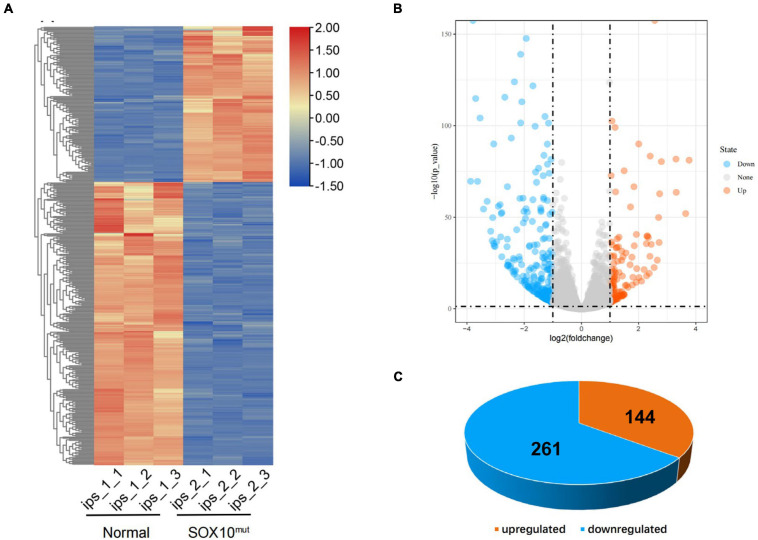
Differentially expressed genes in *SOX10* mutant iPSCs. **(A)** The heatmap showed hierarchical clustering analysis of DEGs in *SOX10* mutant iPSCs. The FPKM values of DEGs were normalized by scale function and compared between the *SOX10* mutant iPSCs and normal control. Red and blue indicate genes with high and low expression levels, respectively. **(B)** Volcano plot showing the expression change of each gene and their significance. Red dots represent the expression of genes in *SOX10* mutant iPSCs significantly up-regulated compared to normal control. Blue dots represent the expression of genes in *SOX10* mutant iPSCs significantly down-regulated compared to normal control. **(C)** Of the DEGs, 144 genes were up-regulated and 261 genes were down-regulated.

To investigate differential gene expression in this WS patient’s iPSCs resulting from *SOX10* mutation, we implemented RNA-Seq analysis of the iPSCs from a normal control. Triplicate RNA samples were isolated from the patient-derived iPSCs and an unrelated control cell line cultured under normal conditions. They were then analyzed using RNA-Seq, and differential gene-expression analysis was performed with DESeq2. A total of 405 genes were found to be differentially expressed between the patient and the pooled control iPSC line based on the differential expression criteria (adjusted *P*-value < 0.05 and | log2 (fold-change) | > 1). A heatmap using the FPKM value of the DEGs was generated and row normalization was executed using scale function (Figure 4A). Among the DEGs, there were 144 genes (35.6%) that displayed significantly augmented expression in the SOX10 mutant iPSCs, while 261 genes (64.4%) showed significantly diminished expression (Figures 4B,C).

**FIGURE 5:**
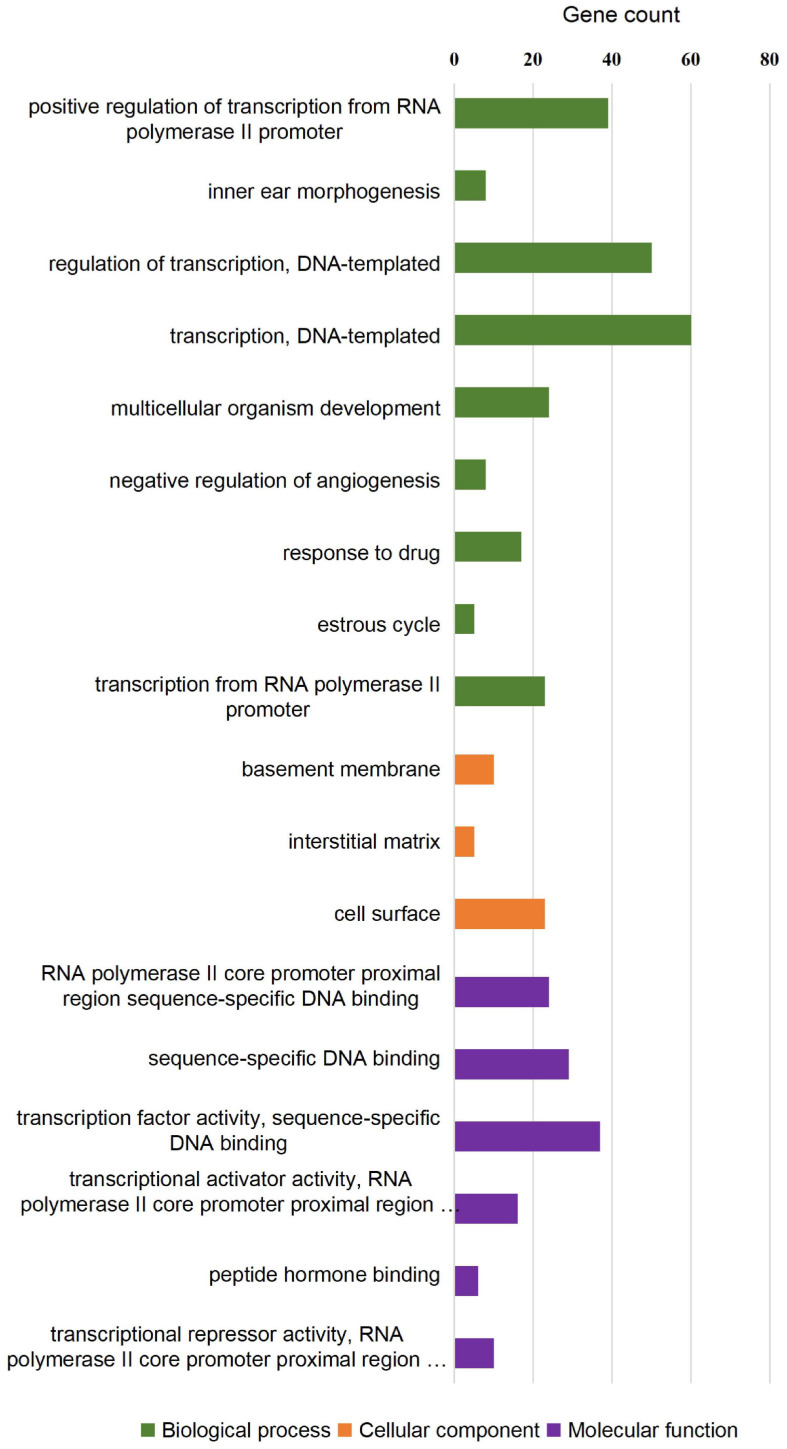
GO enrichment analysis of differentially expressed genes in *SOX10* mutant iPSCs. A total of 18 GO terms were significantly enriched. Nine terms were significantly enriched based on biological process, three terms were significantly enriched based on cellular components, and six terms were significantly enriched based on molecular function.

To investigate whether the DEGs of the *SOX10* mutant iPSCs were enriched in specific functionally related gene groups and signaling pathways, we utilized Gene Ontology (GO) and KEGG (Kyoto Encyclopedia of Genes and Genomes) pathway-enrichment analyses. The significantly enriched GO terms included terms connected to DNA-templated transcription, transcription from RNA polymerase II promoter, multicellular organism development, and negative regulation of angiogenesis (Figure 5). Interestingly, GO enrichment for biological process identified inner ear morphogenesis enriched in the DEGs, and these related genes are listed in Table 1. Additionally, no DEGs of *SOX10* mutant iPSCs were significantly enriched in the KEGG pathways. Altogether, SOX10 deficiency led to subtle transcriptional perturbation with respect to the affected genes and their mRNA levels, and the *SOX10* mutant iPSCs had the ability to undergo morphologic differentiation in a manner similar to those derived from the control iPSCs.

**TABLE 1:** Differentially expressed genes in patient iPSC enriched in inner ear morphogenesis.

No.	Gene symbol	Gene description	Log 2 fold change	Adjusted *P*-value
1	TBX1	T-box transcription factor 1	–3.8729	2.118E-70
2	GBX2	Gastrulation brain homeobox 2	–3.6959	1.398E-115
3	NTN1	Netrin 1	–1.8225	1.016E-34
4	PAX8	Paired box 8	–1.2506	4.331E-06
5	GATA3	GATA binding protein 3	1.1878	1.627E-05
6	SPRY2	Sprouty RTK signaling antagonist 2	–1.0997	2.516E-52
7	HMX2	H6 family homeobox 2	1.0354	2.367E-04
8	CHD7	Chromodomain helicase DNA binding protein 7	1.178	9.746E-100

### Differentiation of Mutated *SOX10* Patient-Derived iPSCs to NCCs

**FIGURE 6:**
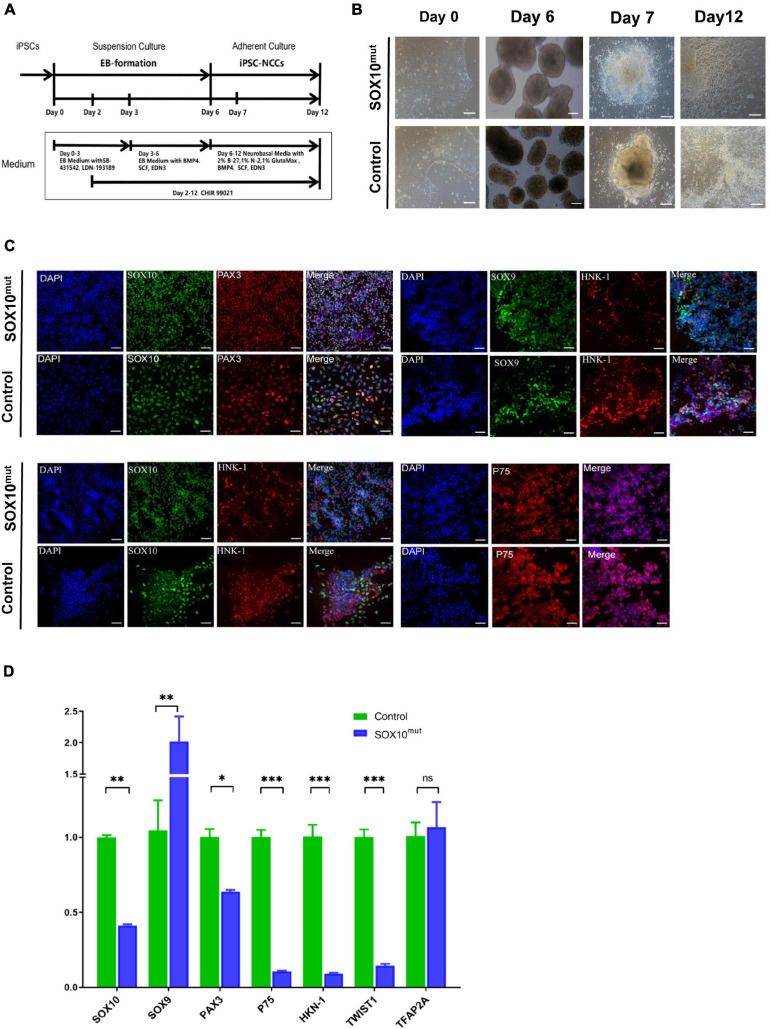
Generation and characterization of *SOX10* mutant iPSC-derived NCCs. **(A)** Schematic of the NCC differentiation protocol timeline. EB Medium: KO-DMEM supplemented with 20% KSR, 1% GlutaMax-I, 1%NEAA. **(B)** Comparison of the cell images of the SOX10 mutant and control iPSC-derived NCC at Days 0, 6, 7, and 12 following differentiation. Bar, 100 μM. **(C)** Immunofluorescence staining shows expression of NCC markers SOX10, PAX3, SOX9, HNK-1, and P75. Bar, 50 μM. **(D)** RT-qPCR for evaluating expression of NCC markers. (^∗^represents *p* < 0.05, ^∗∗^ represents *p* < 0.01, ^∗∗∗^ represent *p* < 0.001 and ns represents no significant).

Following the investigation of the impacted of lowered *SOX10* expression on WS patient-derived iPSCs at the pluripotent stage, we narrowed our study to examine differentiating the iPSCs to iNCCs as a more relevant, disorder-specific cell type. We followed a previously established protocol to differentiate patient-derived and WT iPSCs into neural crest cells (this protocol is described in the “Materials and Methods” sections of these publications, and results showed that activation of the WNT pathway induced neural border genes and neural crest markers that mimicked normal neural crest development) (Chambers et al., 2011; Figure 6A). Our findings indicated an apparent minor delay in neural crest induction in *SOX10* mutant iPSCs. In addition, despite being initially plated at the identical density, less cells were noted in the mutant cultures during 7 d of culture. By day 12, the majority of the areas of the cultures had achieved confluency; in contrast, the patient-derived NCCs were denser (Figure 6B). Immunofluorescence analysis demonstrated that the neural crest (NC) differentiation markers SOX10, SOX9, PAX3, HNK-1, and P75 were expressed in both iNCC cell lines (Figure 6C). We then compared the expression of NC-related genes (*SOX9*, *PAX3*, *HNK-1*, *P75*, *TWIST1*, and *TFAP2A* including *SOX10*) on day 12 of the differentiation process between both types of iNCCs. Under NC induction, the iNCCs derived from *SOX10* mutant iPSCs initiated significant down-regulation of the NC-related genes at the mRNA level except SOX9, compare with control (Figure 6D). Collectively, these observations indicated that SOX10 haploinsufficiency—through the development of NCCs-affected the proliferation and differentiation of NCCs, and reduced their overall pluripotent potential.

### Global Changes in Gene Expression in the WS Patient-Derived iNCCs With the *SOX10* Mutation

**FIGURE 7:**
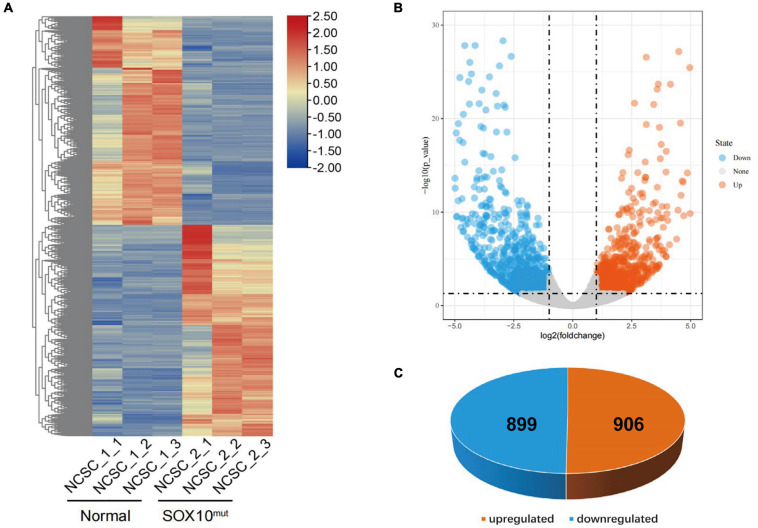
Differentially expressed genes in *SOX10* mutant iNCCs. **(A)** The heatmap showed hierarchical clustering analysis of DEGs in *SOX10* mutant iNCCs. The FPKM values of DEGs were normalized by scale function and compared between the *SOX10* mutant iNCCs and normal control. Red and blue indicate genes with high and low expression levels, respectively. **(B)** Volcano plot showing the expression change of each gene and their significance. Red dots represent the expression of genes in *SOX10* mutant iNCCs significantly upregulated compared to normal control. Blue dots represent the expression of genes in *SOX10* mutant iNCCs significantly downregulated compared to normal control. **(C)** A total of 906 genes were up-regulated and 899 genes were down-regulated among the DEGs.

RNA-Seq analysis was completed in triplicate for iNCCs from the *SOX10* mutant and normal control lines to evaluate cellular differentiation at the gene-expression level between the iNCC lines, and differential gene expression was determined. The methods used for data analysis and sample pooling were the same as the analysis conducted for the iPSCs in order to enable a direct comparison. The heatmap created with the FPKM value for global gene expression indicated that most of the gene-expression patterns differed between the *SOX10* mutant iNCCs and controls (Figure 7A). DESeq2 identified a total of 1805 DEGs (*P*-value < 0.05 and | log2 (fold-change) | > 1), among which 899 genes were downregulated in *SOX10* mutant iNCCs while 906 genes were upregulated in patient iNCCs (Figures 7B,C). The number of DEGs was four times higher in the former relative to the iPSCs, indicating that the *SOX10* mutation had a much stronger impact on the transcriptome in differentiated cells, which corresponded with the tissue-restricted phenotype.

**FIGURE 8:**
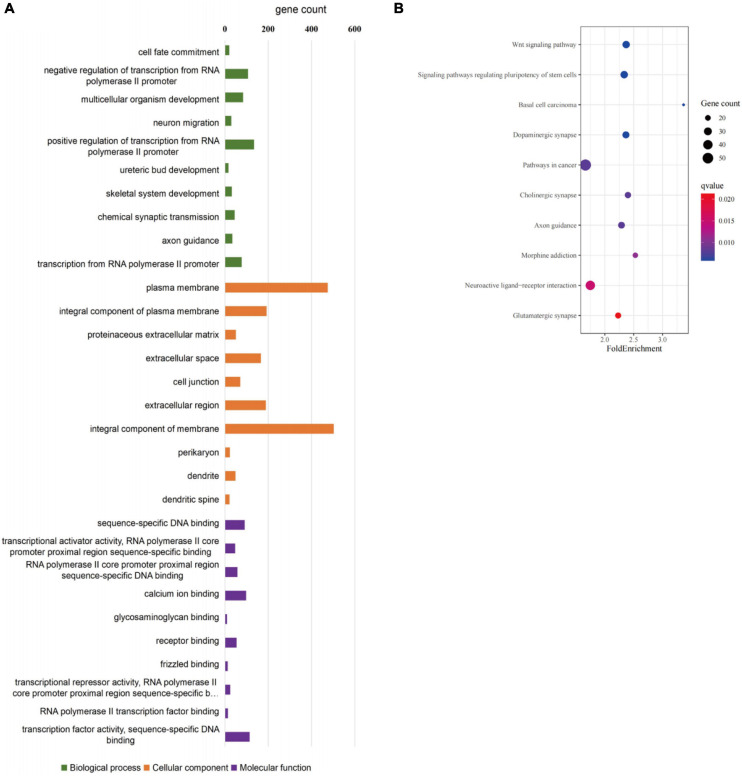
GO enrichment analysis of differentially expressed genes in *SOX10* mutant iNCCs. **(A)** Top 10 most enriched GO terms for biological processes, cellular components, and molecular function. **(B)** Top 10 most enriched KEGG pathway terms are listed.

GO and KEGG pathway-enrichment analyses were performed on all the DEGs to determine whether specific subsets of genes were differentially expressed in the patient iNCCs. In total, 78 GO terms were significantly enriched. Of them, 47 GO terms were significantly enriched to biological process (BP), 19 GO terms were significantly enriched to cellular component (CC), and 12 GO terms were significantly enriched to molecular function (MF) (the top 10 most enriched GO terms for BP, CC, and MF are revealed in Figure 8A). The top 10 most enriched GO terms for BP included multicellular organism development, neuron migration, regulation of transcription from RNA polymerase II promoter, ureteric bud development, skeletal system development, chemical synaptic transmission, and axon guidance. These results indicated that subsets of genes involved in tissues and cell types, including peripheral neurons and glial cells, melanocytes, secretory cells, and cranial skeletal and connective cells, were overrepresented in the DEGs, suggesting that they had strong links to defects in NCC biology and the development of multiple NC-derived systems. GO enrichment for BP also determined enriched functional networks pertaining to inner ear morphogenesis and inner ear development (the related DEGs in these two GO terms are revealed in Tables 2, 3). KEGG pathway analysis identified 17 terms as significantly enriched, and the top-10 KEGG terms included WNT signaling pathway, signaling pathways regulating pluripotency of stem cells, basal cell carcinoma, dopaminergic synapse, pathways in cancer, cholinergic synapse, axon guidance, morphine addiction, neuroactive ligand-receptor interaction, and glutamatergic synapse (Figure 8B).

**TABLE 2:** Differentially expressed genes in patient iNCCs enriched in inner ear morphogenesis.

No.	Gene symbol	Gene description	Log 2 fold change	Adjusted *P*-value
1	GATA3	GATA binding protein 3	–2.5587	1.457E-04
2	USH1G	USH1 protein network component sans	–2.4423	2.017E-02
3	TBX1	T-box transcription factor 1	–2.3788	4.440E-03
4	PRRX1	Paired related homeobox 1	–1.8414	1.492E-03
5	NTN1	Netrin 1	–1.8141	2.494E-04
6	GBX2	Gastrulation brain homeobox 2	–1.7589	1.016E-04
7	ITGA8	Integrin subunit alpha 8	–1.2744	2.697E-02
8	FGF9	Fibroblast growth factor 9	1.4407	1.599E-02
9	COL11A1	Collagen type XI alpha 1 chain	1.5271	8.154E-04
10	TFAP2A	Transcription factor AP-2 alpha	1.648	3.069E-02
11	ZIC1	Zic family member 1	2.0873	3.878E-04
12	MAFB	MAF bZIP transcription factor B	2.6142	4.558E-06
13	POU4F3	POU class 4 homeobox 3	3.2812	2.775E-06
14	NEUROG1	Neurogenin 1	3.7124	1.956E-16

**TABLE 3:** Differentially expressed genes in patient iNCCs enriched in inner ear development.

No	Gene symbol	Gene_description	Log 2 fold change	Adjusted *P*-value
1	LGR5	Leucine rich repeat containing G protein-coupled receptor 5	–5.9869	9.649E-38
2	BMPER	BMP binding endothelial regulator	–5.0578	2.041E-21
3	SHH	Sonic hedgehog signaling molecule	–3.0073	4.947E-05
4	BMP2	Bone morphogenetic protein 2	–2.3362	6.027E-12
5	HOXA1	Homeobox A1	–2.1382	8.994E-03
6	MAF	MAF bZIP transcription factor	1.5757	2.496E-04
7	CYTL1	Cytokine like 1	1.6092	4.109E-02
8	CXCL14	C-X-C motif chemokine ligand 14	1.8429	5.919E-06
9	PLPPR4	Phospholipid phosphatase related 4	1.93	1.373E-02
10	EYA4	EYA transcriptional coactivator and phosphatase 4	1.9746	6.097E-06
11	NEUROD1	Neuronal differentiation 1	3.0814	2.239E-04
12	PHOX2B	Paired like homeobox 2B	3.3696	2.151E-04

**FIGURE 9:**
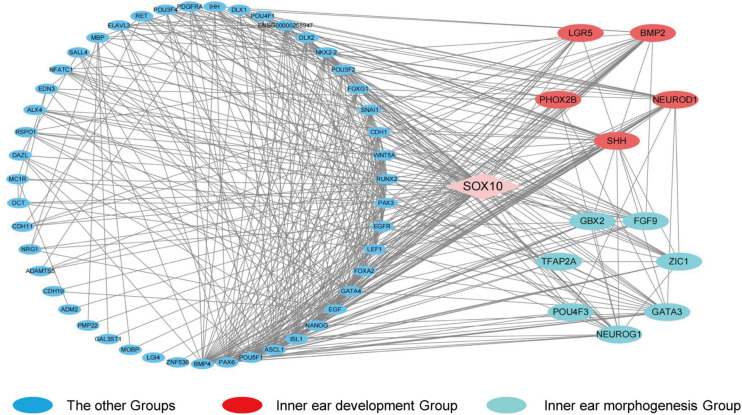
Protein-protein interaction network of differentially expressed genes that interact with SOX10. The diamond represents SOX10. The circles indicate the proteins that interact with SOX10. Red circles represent the genes enriched in the GO term inner ear development, and cyan circles represent the genes enriched in the GO term inner ear morphogenesis. Lines represent the interaction relationship between two proteins.

In order to identify the candidate target gene regulated by SOX10 throughout inner ear development, we examined the genes pertinent to inner ear development in the GO database (GO terms were inner ear development and inner ear morphogenesis) and proteins that interacted with SOX10 in the STRING database. Fifty-nine proteins interacted directly with SOX10 (Figure 9). The gene lists were combined with the DEGs to acquire the target genes connected to inner ear development and morphogenesis. Considering the association between decreased RNA expression and possible SOX10-binding sites allowed us to reduce the list of candidate genes to four: *BMP2*, *LGR5*, *GBX2*, and *GATA3*. The potential SOX10-binding sites in the candidate genes were predicted using the online JASPAR database (Table 4 shows the predicted binding site details).

**TABLE 4:** The SOX10 potential binding sites predicted in the candidate genes.

Name	Score	Relative score	Start	End	Strand	Predicted sequence
BMP2	7.09699	0.91952059	395	400	+	CTGTGT
LGR5	8.90979	0.999999997	669	674	+	CTTTGT
GBX2	8.90979	0.999999997	328	333	+	CTTTGT
GATA3	7.09357	0.919368595	111	116	+	CGTTGT

## Discussion

WS, the most common disorder resulting in syndromic hearing loss (SHL) in the Chinese population, is a genetic disorder with locus heterogeneity and variable expression of clinical characteristics (Zhang et al., 2012; Li et al., 2019). The mechanisms underlying phenotypic variability in WS are still not fully understood (Bondurand et al., 2007; Pingault et al., 2010). SNHL is defined as a pure tone threshold shift of over 25dB, affecting more than 466 million people worldwide. SNHL includes degenerative changes of cochlear hair cells (He et al., 2017, 2021; Liu W. et al., 2019; Zhou et al., 2020; Cheng et al., 2021; Fu et al., 2021), cochlear supporting cells (Lu et al., 2017; Cheng et al., 2019; Tan et al., 2019; Zhang S. et al., 2019; Zhang et al., 2020a; Zhang Y. et al., 2020; Chen et al., 2021), and spiral ganglion neurons (Guo et al., 2016, 2020, 2021; Yan et al., 2018; Liu et al., 2021). Sound is collected and conducted by external and middle ear, then transformed into the electric signals by cochlear hair cells (Wang et al., 2017; Liu Y. et al., 2019; Qi et al., 2019, 2020; Zhang Y. et al., 2020); while spiral ganglion neurons is function as the neural auditory transduction cells (Sun et al., 2016; Guo et al., 2019, 2021; Liu W. et al., 2019; Zhao et al., 2019). The cochlear hair cells are sensitive to aging, acoustic trauma, ototoxic drugs, and environmental or genetic influences (O’Donnell et al., 1988; Zhu et al., 2018; Fang et al., 2019; Jiang et al., 2020; Qian et al., 2020; Lv et al., 2021; Zhang et al., 2021). Previous studies have shown that oxidative stress and cell apoptosis play important roles in hair cell loss (Sun et al., 2014; Yu et al., 2017; Li et al., 2018; Gao et al., 2019; Zhang Y. et al., 2019; Zhang et al., 2020b; Zhong et al., 2020).

SOX10 is a key transcription factor related to the migration and differentiation of NCCs. Mutations in SOX10 result in abnormal pigment distribution and deafness, and are the primary cause of WS (Bondurand and Sham, 2013). SOX10 belongs to the *SOX* family, which features a high-mobility group (HMG) DNA-binding domain. The HMG domain (amino acids 102–181) identifies and binds to the promoter sequence of a target gene and induces conformational modifications in DNA throughout transcriptional regulation (Harris et al., 2010; Schock and LaBonne, 2020).

The non-sense mutation identified in the present study was found in amino acid 112, which is located in the HMG domain (DNA-binding region) and in the predicted nuclear localization signals (NLSs), resulting in a substitution of the guanine in position number 336 (Südbeck and Scherer, 1997). This *SOX10* mutant was first identified by Chaoui et al. (2011) in three independent families, and resulted from two different variations at the nucleotide level: c.336G > A and c.336G > C). The probands were associated with WS2 or PCW/PCWH based on the observed variety of phenotypes. Functional analysis revealed that the p.Met112Ile appeared to possess an increased monomer-binding capacity, leading to reduced binding of the *SOX10* mutant and reduced transactivation capacity toward the target promoter (Chaoui et al., 2011). Nevertheless, the phenotypic differences observed raise the potential for the individual genetic background being influential, which is not uncommon in neurocristopathies (Amiel et al., 2008). Since SOX10 gene is not endogenous expressed at the iPSCs stage, we collected cells at the 12th day of iNCC stage to perform qPCR and WB experiments to analyze the influence of SOX10 mutation (Supplementary Figure 1). The results suggest this mutation caused the decrease of its RNA and protein expression levels in the patient-derived iNCCs, thus it was speculated that it might cause functional changes through insufficient haploid dose.

To the best of our knowledge, this is the first work to document a disease model of iPSCs derived from a patient with WS. There are currently several established SOX10 animal-disease models that entail multiple species (Tachibana et al., 2003; Dutton et al., 2009; Hao et al., 2018). However, there are still many differences between the phenotypes of animal models and those of humans due to the disparities in genetic background, timeline of organ development, and underlying regulatory mechanisms between the species; it is therefore still difficult to accurately recapitulate human abnormalities such as WS in animals. Because iPSCs can differentiate into a vast array of cell types, the present system provides a powerful method to elucidate the disease mechanisms and explore potential therapeutic interventions so as to improve the well-being of patients (Chen et al., 2019; Zhang et al., 2020a).

In the current work, we generated a human cell model for WS with iPSCs harboring a *SOX10* mutation, and differentiated these iPSCs into NCCs as a specific and disease-relevant system that could be used to investigate WS *in vitro*. WS patient-derived fibroblasts were reprogrammed into *SOX10*-mutant iPSCs based on the Yamanaka method (Takahashi and Yamanaka, 2006). The *SOX10*-mutant iPSCs generated in this study could then be further cultured with relatively high efficiency and showed pluripotential characteristics, including pluripotency marker expression and the potential for teratoma formation, suggesting that the mutation in *SOX10* did not directly affect the induction and expression of the iPSCs.

In contrast to the normal control, the idiopathic *SOX10* mutant iPSCs exhibited lowered efficiency in NCC induction *in vitro* and defects in the expression of key genes in NCC specification. Interestingly, unlike other NCC markers, the expression of SOX9 was increased in the qRT-PCR of SOX10 mutated iNCC cells compared with the normal group. The SOX transcription group consists of SOX9 and SOX10, and they have a common bipartite transactivation mechanism. In addition, they share some overlap in biological functions (Haseeb and Lefebvre, 2019). The decrease of SOX10 expression may lead to the compensatory increase of SOX9 expression. Relative to the normal control, the transcriptomic analysis of *SOX10* mutant iPSCs revealed an overrepresentation of genes in the embryologic development of the tissues principally impacted in WS, such as pigmentation and skeletal and neuronal development. We identified a total of 1,805 DEGs, of which 899 (49.8%) were down-regulated. These results suggest that SOX10 mutation have a wide range of effects on the transcriptome, and that the target genes involved in the biological process are enriched, suggesting that SOX10 mutation have an impact on the proliferation and differentiation potential of NCCs, which is also in accordance with previous studies (Mollaaghababa and Pavan, 2003; Haldin and LaBonne, 2010; Schock and LaBonne, 2020).

We noted that our analyses converged, suggesting potential mechanisms of inner ear development as the proband showed conspicuous bilateral inner ear malformations. In previous studies, researchers demonstrated that, rather than resulting from an NCC defect, inner ear malformations were directly induced by a *SOX10* mutation by causing endolymphatic collapse and other abnormalities in the organ of Corti (Elmaleh-Bergès et al., 2013; Locher et al., 2015; Hao et al., 2018). However, some other researchers have explored the exact contributions of neural crest lineages to the neurosensory components of the inner ear, offering an important basis for investigating the potential NC origins of the inner ear (Freyer et al., 2011). After examining the GO and KEGG pathway enrichment analyses for the DEGs, we determined that biological processes focused on inner ear development and morphogenesis in both iPSCs and iNCCs, suggesting that the mutation in *SOX10* may have caused the inner ear malformation in this WS patient.

While a growing body of evidence has revealed that *SOX10* mutations can cause defects of the inner ear in humans, the target genes and pathways regulated by *SOX10* that are involved in inner ear development have yet to be completely elucidated (Elmaleh-Bergès et al., 2013; Wakaoka et al., 2013; Song et al., 2016; Xu et al., 2016). We additionally performed a cluster analysis to screen the PPI network pertaining to *SOX10*, and it revealed four candidate genes that may be regulated by *SOX10* during the development of the inner ear: *BMP2*, *LGR5*, *GBX2*, and *GATA3*.

*BMP2* (bone morphogenetic protein 2), a member of the transforming growth factor-beta (TGF-β) superfamily, possesses crucial functions in developmental processes, including cardiogenesis, digit apoptosis, somite formation, neuronal growth, and musculoskeletal development (Schlange et al., 2000; Benavente et al., 2012; Christen et al., 2012; Gámez et al., 2013). As mentioned in a literature review, *BMP2* plays a crucial role in the formation of three semicircular canals during inner ear development (Hwang et al., 2019). The otic-specific knockout of *Bmp2* caused the lack of all semicircular canals in a mouse model (Hwang et al., 2010). Additionally, *bmp2b* was also shown to be necessary for maintaining canal structures in zebrafish, as mutant *bmp2b* zebrafish lacked canals, which is similar to the mouse mutants. *Bmp2* is expressed in highly conserved patterns in the canals’ genesis zones near the cristae, as well as in the epithelium of the developing canals (Hammond et al., 2009). Moreover, *BMP2* takes part in the regulation of NCC proliferation, migration, and differentiation—mimicking the expression patterns of the *SOX10* gene. Previous studies showed that *BMP*2 is also required for enteric nervous system development. The expression of *BMP2* is significantly attenuated in Hirschsprung’s disease patients—which results from defects in NCCs colonizing the intestines—and leads to an absence of enteric ganglia in the colon (Huang et al., 2019). In addition, *BMP2* selectively targets and stimulates tyrosinase (TYR) gene expression and melanogenesis in differentiated melanocytes. It has been reported that *BMP2* treatment of neural crest cells increases melanogenesis by encouraging the synthesis of melanin and the *BMP2* response-element localized upstream from the *TYR* transcriptional start site (Bilodeau et al., 2001). *SOX9* also encourages the expression of BMP2 by binding directly to the *BMP2* promoter, promoting its transcription (Xiao et al., 2019). Therefore, we suggest that *SOX9* and *SOX10* comprise a *SOX*-transcription group and share a bipartite transactivation mechanism that implicates the direct regulation of *BMP2* by *SOX10* (Haseeb and Lefebvre, 2019).

*LGR5* (leucine-rich repeat-containing G-protein coupled receptor 5) is a target gene of the Wnt pathway and a known indicator of endogenous stem cells in rapidly proliferating organs (Barker et al., 2007; Jaks et al., 2008). In addition, *LGR5* plays key roles in embryonic development and in the regeneration and preservation of adult stem cells (Chaoui et al., 2011). In a pattern emulating that of *SOX10*, *LGR5* is widely expressed in NCCs at early stages of embryonic development (Boddupally et al., 2016). *LGR5* is expressed in the apical poles of the sensory epithelium of the cochlear duct and vestibular end organs, and has limited expression in the hair cells of the organ of Corti during early embryonic development (Chai et al., 2011). Previous research has demonstrated that *Lgr5* + cochlear supporting cells (SCs) can regenerate hair cells (HCs) via direct differentiation and mitotic regeneration (Wang et al., 2015). Differentially expressed genes can be found between Lgr5^+^ progenitors and *Lgr5*-SCs that may regulate the proliferation of the *Lgr5*^+^ progenitors and the regenerative capacity of HCs (Cheng et al., 2017).

*GBX2* (gastrulation brain homeobox 2) encodes a DNA-binding transcription factor that plays critical roles in embryogenesis. Several studies have concluded that *GBX2* is needed for the development of the inner ear, especially during the initial formation of the otic placode (Miyazaki et al., 2006; Steventon et al., 2012, 2016). The *Gbx2*^–/–^ mouse displays several inner ear abnormalities, ranging from local malformation to a complete loss of vestibular and cochlear inner ear structures—including the absence of semicircular canals, malformed saccule, and cochlear duct (Lin et al., 2005). Several current studies have also depicted pivotal parts *GBX2* plays in the induction, migration, and patterning of NCCs by impacting multiple facets of NC development (Li et al., 2009; Chervenak et al., 2014; Roeseler et al., 2020). The loss of *GBX2* function also modulates the Slit/Robo-signaling pathway, leading to abnormal NCC migration and abnormalities that as similar to those in congenital diseases, such as DiGeorge syndrome and in craniofacial malformations (Byrd and Meyers, 2005; Calmont et al., 2009).

*GATA3* belongs to the GATA family of transcription factors and is a key regulator of auditory system development (Karis et al., 2001; Appler and Goodrich, 2011). Its expression is found in virtually all auditory cell types (Rivolta and Holley, 1998; Lawoko-Kerali et al., 2002; Milo et al., 2009). In early inner ear development from thembryonic otic placode, *GATA3* regulates the signaling of prosensory genes in a dynamic fashion and at the same time, it directs the differentiation of cochlear neurosensory cells (Duncan and Fritzsch, 2013; Moriguchi et al., 2018). Further studies have provided evidence that *GATA3* is also crucial for the coordinated maturation of sensory hair cells and their innervation (Bardhan et al., 2019). In humans, the expression of *GATA3* is localized to the cochlear duct and the spiral ganglion between weeks 8 and 12 of gestation (Roccio et al., 2018); the loss of GATA3 in inner hair cells leads to hearing loss and accounts for some of the deafness connected to hypoparathyroidism and renal anomaly (HDR) syndrome (Van Esch et al., 2000; Martins et al., 2018). Researchers have also demonstrated that *GATA3* plays critical roles in neural crest cell development and neuronal differentiation in some cranial neural crest derivatives (George et al., 1994; Lieuw et al., 1997; Lakshmanan et al., 1999).

**FIGURE 10:**
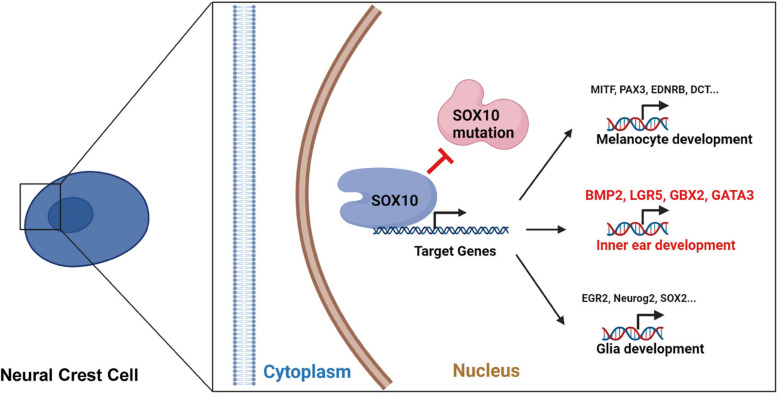
The schematic diagram for the *SOX10*-regulated transcription of certain target genes during development and differentiation in neural crest cell.

In conclusion, in this work, we created a WS human iPSC model with a *SOX10* mutation, and allowed the differentiation of iPSC into NCCs. Relative to normal controls, the WS patient-specific iPSCs had a poor response to NCC induction *in vitro* and a compromised differentiation potential in regard to the NCCs’ fate. Transcriptional perturbation in NCC differentiation in this model was revealed through the intensive analysis of high-throughput RNA-seq results. In addition, we identified numerous candidate genes that are highly likely to be related to inner ear malformation in WS patients with a *SOX10* mutation (Figure 10). Because the molecular mechanisms underlying the effect of *SOX10* on inner ear development have not been fully elucidated, our research offers a rich context for investigating the molecular etiology of WS in regard to inner ear malformations. Nevertheless, additional research is necessary in order to verify the part that the determined target genes and their pathways have in triggering inner ear malformations.

## Data Availability Statement

The datasets presented in this study can be found in online repositories. The names of the repository/repositories and accession number(s) can be found below: https://www.ncbi.nlm.nih.gov/geo/query/acc.cgi?acc=GSE176101, accession: GSE176101.

## Ethics Statement

The studies involving human participants were reviewed and approved by the Ethics Committee of Xiangya Hospital Central South University. Written informed consent to participate in this study was provided by the participants’ legal guardian/next of kin. The animal study was reviewed and approved by The Ethics Committee of Xiangya Hospital Central South University. Written informed consent was obtained from the individual(s) for the publication of any potentially identifiable images or data included in this article.

## Author Contributions

JS, CH, and YF conceived and designed the study. JW performed the most of the experiments. YB and LM analyzed the related data. YL contributed to the generation of iPSC lines. XC, CH, and LYM were responsible for the patient recruitment and obtaining consent for all of the patients, and clinical sample collection. JW and JS wrote the manuscript. CH and YF supervised the study. All authors approved the final version.

## Conflict of Interest

The authors declare that the research was conducted in the absence of any commercial or financial relationships that could be construed as a potential conflict of interest.

## Publisher’s Note

All claims expressed in this article are solely those of the authors and do not necessarily represent those of their affiliated organizations, or those of the publisher, the editors and the reviewers. Any product that may be evaluated in this article, or claim that may be made by its manufacturer, is not guaranteed or endorsed by the publisher.

## Footnotes

1
http://www.networkanalyst.ca

2
https://string-db.org/
