# Supplementary figures and images for: A Model of Waardenburg Syndrome Using Patient-Derived iPSCs With a SOX10 Mutation Displays Compromised Maturation and Function of the Neural Crest That Involves Inner Ear Development
Source: Front Cell Dev Biol. 2021 Aug 6;9:720858. doi: 10.3389/fcell.2021.720858 (PMC8379019; doi:10.3389/fcell.2021.720858)

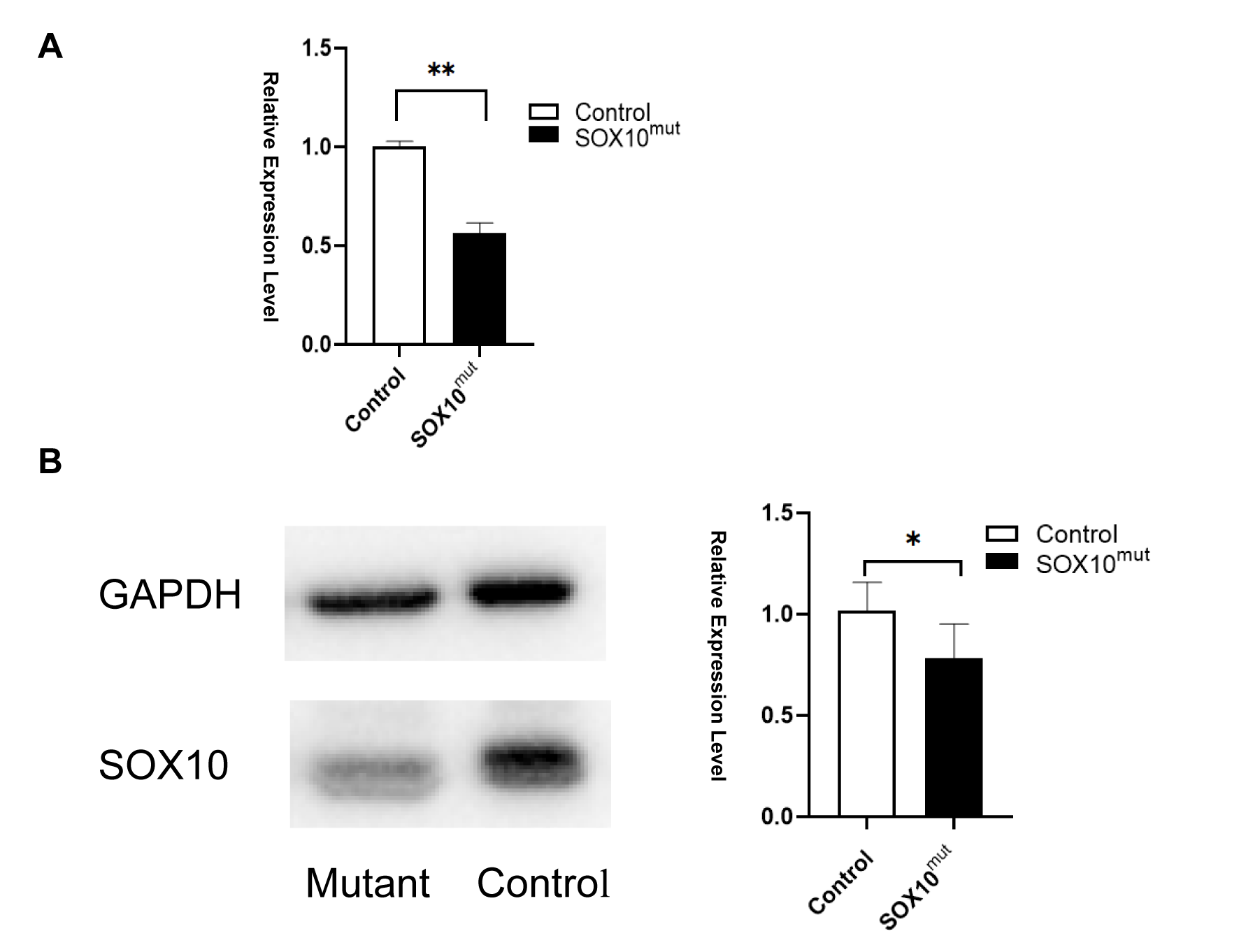

Supplement: Supplementary file 1 [file Image_1.TIF]
