# Supplementary material for: A Model of Waardenburg Syndrome Using Patient-Derived iPSCs With a SOX10 Mutation Displays Compromised Maturation and Function of the Neural Crest That Involves Inner Ear Development
Source: Front Cell Dev Biol. 2021 Aug 6;9:720858. doi: 10.3389/fcell.2021.720858 (PMC8379019; doi:10.3389/fcell.2021.720858)
Supplement: Supplementary file 2 [file Table_1.DOCX]

**Supplementary Files**

**Supplementary Table 1. Reagents details**

| **Primers** | **Targets** | **Forward/Reverse primer (5′−3′)** |
| --- | --- | --- |
| **Targeted mutation analysis** | SOX10 | AAGGTCAAGAAGGAGCAGCA/  TACTGGTCCGCGAGCTTC |
| **Pluripotency Marker (RT-PCR)** | OCT4 | CCTCACTTCACTGCACTGTA/  CAGGTTTTCTTTCCCTAGCT |
|  | NANOG | TGAACCTCAGCTACAAACAG/  TGGTGGTAGGAAGAGTAAAG |
|  | SOX2 | CCCAGCAGACTTCACATGT/  CCTCCCATTTCCCTCGTTTT |
| **Neural crest cell Marker (RT-PCR)** | SOX10 | AGCCCAGGTGAAGACAGAGA/  ATAGGGTCCTGAGGGCTGAT |
|  | PAX3 | AGCCGCATCCTGAGAAGTAA/  CTTCATCTGATTGGGGTGCT |
|  | SOX9 | AGTGGGTAATGCGCTTGGATAGGT/  CGAAGATGGCCGAGATGATCCTAA |
|  | P75 | GTGGGACAGAGTCTGGGTGT/  AAGGAGGGGAGGTGATAGGA |
|  | HNK-1 | ACGACGACAACACCTACAGC/  ACCTCGCAGCTTGAAGTAGG |
|  | PAX6 | ACCCATTATCCAGATGTGTTTGCCCGAG/  ATGGTGAAGCTGGGCATAGGCGGCAG |
|  | TFAP2A | TCCCTGTCCAAGTCCAACAGCAAT/  AAATTCGGTTTCGCACACGTACCC |
|  | TWIST1 | GCCGGAGACCTAGATGTCATT/  CCACGCCCTGTTTCTTTGAA |
|  | GAPDH | CAAGGTCATCCATGACAACTTTG/  GTCCACCACCCTGTTGCTGTA G |

**Supplementary Table 2. Reagents details**

|  | **Antibody** | **Dilution** | **Company and Cat #** |
| --- | --- | --- | --- |
| **Pluripotency Markers** | Rabbit anti-OCT3/4 | 1:400 | Abcam, ab109884 |
|  | Rabbit anti-NANOG | 1:200 | Abcam, ab109884 |
|  | Mouse anti-SSEA-4 | 1:400 | Abcam, ab109884 |
|  | Mouse anti-TRA-1-60 | 1:500 | Santa Cruz, sc21705 |
|  | Mouse anti-SOX2 | 1:500 | Santa Cruz, sc365823 |
| **Neural crest cell Markers** | Rabbit anti-SXO10 | 1:500 | Abcam, ab155279 |
|  | Rabbit anti-P75 | 1:300 | Abcam, ab52987 |
|  | Rabbit anti-SXO9 | 1:500 | Abcam, ab185966 |
|  | Mouse anti-PAX3 | 1:300 | R&D, MAB2457 |
|  | Mouse anti-HNK-1 | 1:400 | Sigma, C6680 |
| **Secondary antibodies** | Donkey anti-Rabbit IgG Alexa Fluor 488 | 1:600 | Yeasen, 34206ES60 |
|  | Goat anti-Mouse IgG Alexa Fluor 594 | 1:800 | Yeasen, 33212ES60 |
|  | Goat anti-Rabbit IgG Alexa Fluor 546 | 1:1000 | Thermo Fisher, A-11035 |
