# Supplementary material for: A Model of Waardenburg Syndrome Using Patient-Derived iPSCs With a SOX10 Mutation Displays Compromised Maturation and Function of the Neural Crest That Involves Inner Ear Development
Source: Front Cell Dev Biol. 2021 Aug 6;9:720858. doi: 10.3389/fcell.2021.720858 (PMC8379019; doi:10.3389/fcell.2021.720858)
Supplement: Supplementary file 3 [file Table_2.DOCX]

**Supplementary Figure 1.**

**The expression of mutant SOX10 was down-regulated in iNCCs.**

1. RT-qPCR for evaluating expression of mutant SOX10**.** **B)** Western blot analysis showing the effect of mutant SOX10 protein expression. GAPDH is used as a loading control.(*represents p < 0.05）
